# Nef-specific CD45RA+ CD8+ T cells secreting MIP-1β but not IFN-γ are associated with nonprogressive HIV-1 infection

**DOI:** 10.1186/1742-6405-7-20

**Published:** 2010-07-02

**Authors:** Claudia J Dembek, Sarah Kutscher, Silvia Heltai, Simone Allgayer, Priscilla Biswas, Silvia Ghezzi, Elisa Vicenzi, Dieter Hoffmann, Peter Reitmeir, Giuseppe Tambussi, Johannes R Bogner, Paolo Lusso, Hans-J Stellbrink, Elena Santagostino, Thomas Vollbrecht, Frank D Goebel, Ulrike Protzer, Rika Draenert, Marco Tinelli, Guido Poli, Volker Erfle, Mauro Malnati, Antonio Cosma

**Affiliations:** 1 Institute of Virology, Helmholtz Zentrum München - German Research Center for Environmental Health, 85764 Neuherberg, Germany; 2 Clinical Cooperation Group "Immune Monitoring", Helmholtz Zentrum München - German Research Center for Environmental Health, 85764 Neuherberg, Germany; 3 Institute of Health Economics and Health Care Management, Helmholtz Zentrum München - German Research Center for Environmental Health, 85764 Neuherberg, Germany; 4 Human Virology Unit, San Raffaele Scientific Institute, 20132 Milan, Italy; 5 AIDS Immunopathogenesis Unit, San Raffaele Scientific Institute, 20132 Milan, Italy; 6 Viral Pathogens and Biosafety Unit, San Raffaele Scientific Institute, 20132 Milan, Italy; 7 Laboratory of Clinical Immunology, San Raffaele Scientific Institute, 20132 Milan, Italy; 8 Department of Infectious Diseases, San Raffaele Scientific Institute, 20132 Milan, Italy; 9 Institute of Virology, Technische Universität München, 81675 Munich, Germany; 10 Department of Infectious Diseases, Medizinische Poliklinik, Ludwig Maximilians Universität, 80336 Munich, Germany; 11 Infektionsmedizinisches Centrum Hamburg ICH, 20146 Hamburg, Germany; 12 "A. Bianchi Bonomi" Haemophilia and Thrombosis Center, University of Milan, 20122 Milan, Italy; 13 Division of Infectious and Tropical Diseases, Hospital of Lodi, 26866 Lodi, Italy; 14 Vita-Salute San Raffaele University, School of Medicine, 20132, Milano, Italy

## Abstract

**Background:**

Long-term survival of HIV-1 infected individuals is usually achieved by continuous administration of combination antiretroviral therapy (ART). An exception to this scenario is represented by HIV-1 infected nonprogressors (NP) which maintain relatively high circulating CD4+ T cells without clinical symptoms for several years in the absence of ART. Several lines of evidence indicate an important role of the T-cell response in the modulation of HIV-1 infection during the acute and chronic phase of the disease.

**Results:**

We analyzed the functional and the differentiation phenotype of Nef- and Tat-specific CD8+ T cells in a cohort of HIV-1 infected NP in comparison to progressors, ART-treated seropositive individuals and individuals undergoing a single cycle of ART interruption. We observed that a distinctive feature of NP is the presence of Nef-specific CD45RA+ CD8+ T cells secreting MIP-1beta but not IFN-gamma. This population was present in 7 out of 11 NP. CD45RA+ IFN-gamma^neg ^MIP-1beta+ CD8+ T cells were not detected in HIV-1 infected individuals under ART or withdrawing from ART and experiencing a rebounding viral replication. In addition, we detected Nef-specific CD45RA+ IFN-gamma^neg ^MIP-1beta+ CD8+ T cells in only 1 out of 10 HIV-1 infected individuals with untreated progressive disease.

**Conclusion:**

The novel antigen-specific CD45RA+ IFN-gamma^neg ^MIP-1beta+ CD8+ T cell population represents a new candidate marker of long-term natural control of HIV-1 disease progression and a relevant functional T-cell subset in the evaluation of the immune responses induced by candidate HIV-1 vaccines.

## Background

Increasing evidence in humans and in nonhuman primate models of HIV-1 infection indicates that CD8+ T cells play a direct role in controlling or limiting HIV-1 replication. CD8+ T-cell depletion during acute [1] or chronic [2] SIV infection is associated with a significant increase in viral load. CD8+ T cells exert a strong selective pressure on SIV [3] and HIV-1 [4], whereas expression of particular MHC class I alleles correlates with delayed disease progression in HIV-1 infected individuals [5,6]. However, long-term control of HIV-1 disease is achieved only in a minority of infected individuals, and the mechanisms by which CD8+ T cells contain HIV-1 replication remain unclear. Indeed, high frequencies of IFN-γ producing HIV-1-specific CD8+ T cells have been found in nonprogressors (NP) as well as in untreated HIV-1 infected individual with progressive disease [7]. The magnitude of the specific cellular immune response in antiretroviral therapy (ART)-naive individuals generally correlates with viral load [8-10]. The introduction of polychromatic flow cytometry technology uncovered a high level of complexity in terms of CD8+ T-cell functional and differentiation markers, and it is now well accepted that the sole evaluation of IFN-γ provides limited information on the quality of antigen-specific CD8+ T-cell responses [11,12]. Indeed, recent studies demonstrated that polyfunctional HIV-1-specific CD8+ T cells are associated with nonprogressive HIV-1 infection [13]. In addition, measurement of IFN-γ secretion in combination with the differentiation markers CCR7 and CD45RA revealed an enrichment of HIV-1-specific, fully differentiated effector cells in NP [14] and in individuals with early infection and low viral set point thereafter [15]. In these studies, ART naive individuals with detectable viremia were chosen as controls and compared to NP with low or undetectable viremia. Thus, it was not clear whether these HIV-1-specific T-cell populations were the cause or the consequence of the low viremia and of the nonprogressive status. Interestingly, a successive longitudinal study on a cohort of individuals starting ART and followed for more than two years showed the emergence of polyfunctional CD8+ T cells after prolonged suppression of viremia [16], suggesting that polyfunctional CD8+ T cells are lost under the condition of high antigen exposure and recovered or maintained when the antigen level is low.

In order to improve our understanding of the relationship between cellular immune response and nonprogressive HIV-1 infection, we analyzed the CD8+ T-cell response in the peripheral blood compartment of HIV-1 infected individuals with different histories of infection. Eleven NP were compared to 10 progressors (PR) with unrestricted control of viral replication. All NP and PR had not received ART before. In addition, we analyzed 23 ART-treated patients in whom HIV-1 replication is pharmacologically controlled and the role of the immune system is less relevant. Finally, we characterized the immune response of 6 ART-treated patients who interrupted the assumption of ART investigating the effect of rebounding virus replication on the HIV-1-specific CD8+ T cell responses. We focused on the role of specific CD8+ T cells with respect to the non-structural HIV-1 proteins Nef and Tat. Indeed, these two nonstructural proteins are known to strongly influence HIV-1 replication, pathogenicity and the host immune response [17,18]. Since previous studies associated the presence of polyfunctional [13] and terminally differentiated [14,15,19] CD8+ T cells with the capacity to control viral replication, we coupled the simultaneous detection by intracellular staining of 4 functional markers, i.e. IFN-γ, IL-2, CD154 and MIP-1β with the expression of CD45RA. The use of CD45RA allowed the discrimination between antigen-specific terminally-differentiated effector CD8+ T cells (CD45RA+), also termed T_EMRA_, and the precursor CD45RA^neg ^memory CD8+ T cells, subdivided into central memory, T_CM _and effector memory, T_EM_. By applying this experimental setting, we identified a population of HIV-1-specific CD8+ T cells which is significantly associated with the NP cohort, completely absent in the cohort of ART-treated patients and not related to the levels of viral replication.

## Results

### Nef-specific CD45RA+ IFN-γ^neg ^IL-2^neg ^MIP-1β+ CD8+ T cells are a specific signature of NP

**Figure 1:**
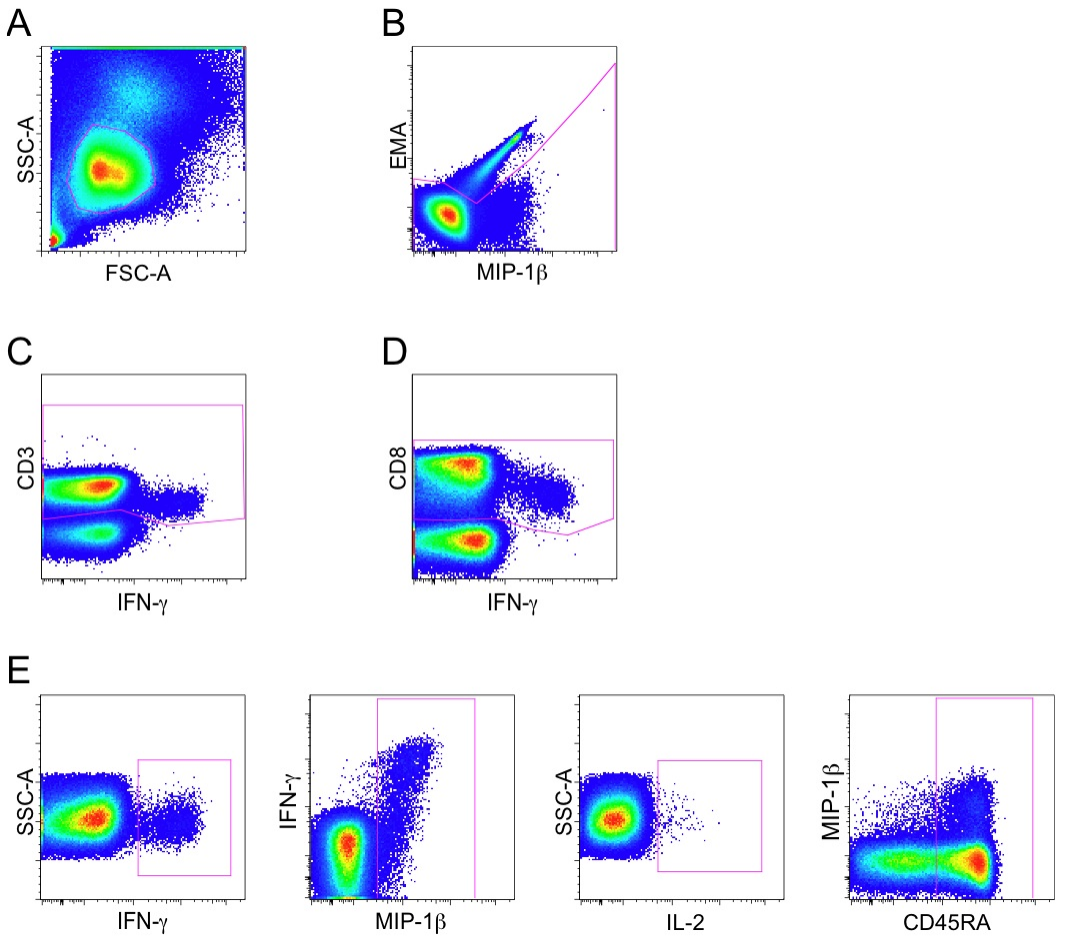
**Gating strategy for the definition of responding and CD45RA+ CD8+ T cells**. First, lymphocytes were gated based on FSC versus SSC plot (**A**), followed by exclusion of dead cells by EMA staining (**B**). As representatively shown, we gated for CD3+ cells on all functional markers to account for CD3 downregulation in antigen specific responding T-cells and combined these gates with the Boolean operator "OR" to obtain the CD3+ cell population (**C**). As representatively shown, we gated for CD8+ cells on all functional markers to account for CD8 downregulation in antigen specific responding T-cells and combined these gates with the Boolean operator "OR" to obtain the CD8+ cell population (**D**). CD4+ T cells were excluded from the CD8+ T-cell population. Once the CD8+ T-cell population was defined, cells positive for IFN-γ, MIP-1β, IL-2 and CD45RA were separately identified by using 4 different plots in which the axis were chosen to provide the best discrimination between positive and negative events (**E**). The complete gating strategy is shown for patient NP13. Selection of positive cells for the functional markers was done by comparison with a mock-stimulated sample.

**Figure 2:**
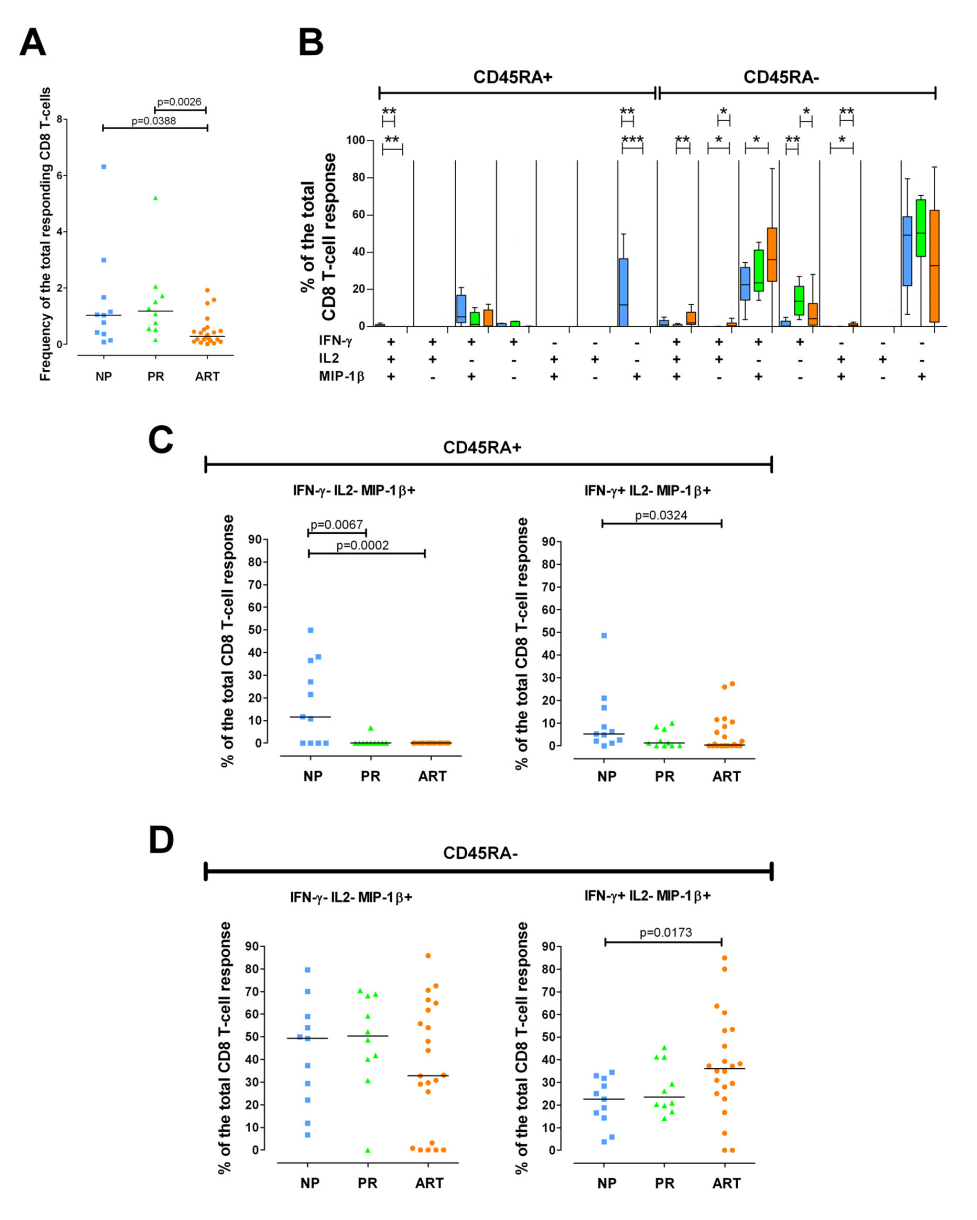
**HIV-1-Nef-specific CD8+ T cell response**. HIV-1-Nef-specific CD8+ T cell response in 11 NP (blue), 10 PR (green) and 22 ART-treated individuals (orange). Nef-specific responses were not analyzed in subject ART07. (**A**) Frequency of the total Nef-specific CD8+ T cells in NP, PR and ART-treated individuals. (**B**) Quality of the Nef-specific response. The graph is divided into the CD45RA+ (left part) and the CD45RA^neg ^(right part) CD8+ T-cell populations. All the possible combinations of the responses are shown on the x-axis for NP, PR and ART-treated individuals. Tukey boxes and whisker plots are shown. Significant differences are noted above the graph: (*) p < 0.05, (**) p < 0.01 and (***) p < 0.001. Individual data point representation of selected HIV-1-Nef-specific CD45RA+ (**C**) or CD45RA^neg ^**(D**) CD8+ T-cell populations. Percentages of the total responses are shown for IFN-γ^neg ^IL-2^neg ^MIP-1β+ CD8+ T cells on the left and for IFN-γ+ IL-2^neg ^MIP-1β+ CD8+ T cells on the right. In all graphs, medians are represented by horizontal bars.

Nef- and Tat-specific CD8+ T-cell responses were analyzed by multicolor flow cytometry in a cohort of NP and compared to responses observed in PR and ART-treated patients (Table 1). Following stimulation with pools of overlapping peptides, we simultaneously measured the expression of CD45RA and the production of IFN-γ, IL-2, CD154 and MIP-1β. The gating strategy is shown in Figure 1. We detected Nef-specific CD8+ T-cell responses in all individuals. However, in 5 ART-treated individuals and 1 NP, responses were slightly above the threshold level. NP and PR showed higher frequencies of total Nef-specific CD8+ T cells when compared to ART-treated patients (Figure 2A). Correlation analysis showed that there was no statistically significant correlation between frequencies of total responses and plasma viral load in the three cohorts analyzed (data not shown). Nevertheless, subject NP13 that showed the highest plasma viral load, had also the highest Nef-specific response.

**Table 1:** Patient characteristics

Patient	Years of known seropositivity	Years of ART	CD4 counts (cells/μl)	CD8 counts (cells/μl)	HIV-1 RNA Copies/ml of Plasma
NP06	19	-	626	3341	214
NP08	14	-	466	957	720
NP09	17	-	421	864	1100
NP11	19	-	274	747	1800
NP13	13	-	502	967	10756
NP14	20	-	461	464	488
NP15	20	-	532	991	8954
NP16	21	-	1042	1091	50
NP17	23	-	924	1030	1083
NP18	25	-	511	500	900
NP19	9	-	842	914	196
PR03	6	-	466	2322	316212
PR05	0	-	208	237	610000
PR11	6	-	214	808	>500000
PR12	2	-	457	1162	489978
PR25	1	-	474	1180	268919
PR34	10	-	326	2628	113164
PR36	19	-	132	834	>500000
PR77	9	-	226	1265	105488
PR86	1	-	406	2448	99402
PR95	0	-	280	1186	123818
ART12	23	4	413	740	36600
ART14	23	8	470	1926	<40
ART15	22	9	345	478	<40
ART16	23	9	609	888	<40
ART01^a^	7	7	969	789	<50
ART02^a^	10	6	609	1033	<50
ART03^a^	6	6	347	1542	<50
ART04^a^	4	4	334	455	<50
ART05^a^	5	5	688	643	<50
ART06^a^	6	6	455	1378	<50
ART07	23	15	532	840	<50
ART08	23	16	229	833	<50
ART09	13	13	351	281	<50
ART10	22	11	777	850	<40
ART11	24	15	271	1350	3362
ART21	15	4	401	1893	<50
ART23	17	5	954	2403	13965
ART25	16	6	593	1671	<50
ART26	9	4	715	846	<50
ART27	6	5	488	851	<50
ART28	7	6	708	NA	<50
ART30	17	13	488	460	<50
ART31	2	2	698	NA	<50

^a ^These patients were later enrolled in a single cycle therapy interruption study.

To assess the quality of the specific responses, we calculated all possible combinations of IFN-γ, IL-2, MIP-1β and CD154 expression in the responding CD45RA+ and CD45RA^neg ^CD8+ T cells. The staining panel was originally designed as routine immune-assay to evaluate simultaneously CD4+ and CD8+ T cell responses, and for this reason includes the measurement of CD154 [20]. As expected and in agreement with previous reports [21], we did not find CD8+ T cells expressing CD154, and therefore this marker was excluded from the analysis of the quality of the CD8+ T cell response. Nef-specific responses were mainly composed of CD45RA^neg ^CD8+ T cells expressing MIP-1β or MIP-1β and IFN-γ (Figure 2B and 2D). The analysis of the quality of the CD8+ T cell response revealed significant differences between the three cohorts (Figure 2B). Highly statistically significant differences (p < 0.01) among the proportion of responding CD8+ T cells in NP, PR and ART-treated patients were found in CD45RA+ IFN-γ+ IL-2+ MIP-1β+, CD45RA+ IFN-γ^neg ^IL-2^neg ^MIP-1β+, CD45RA^neg ^IFN-γ+ IL-2+ MIP-1β+, CD45RA^neg ^IFN-γ+ IL-2^neg ^MIP-1β^neg ^and CD45RA^neg ^IFN-γ^neg ^IL-2+ MIP-1β+ CD8+ T-cell populations. The proportion of polyfunctional (IFN-γ+ IL-2+ MIP-1β+) Nef-specific CD45RA+ CD8+ T cells was significantly higher in NP (median 0.79%; range 0 to 1.90%) than in PR (median 0%; range 0 to 0.03%) or ART-treated individuals (median 0%; range 0 to 1.69%), whereas the proportion of polyfunctional CD45RA^neg ^CD8+ T cells was significantly higher in ART-treated subjects (median 2.04%; range 0 to 11.9%) than in PR (median 0.18%; range 0 to 1.69%). On the other hand, monofunctional Nef-specific CD45RA^neg ^IFN-γ+ IL-2^neg ^MIP-1β^neg ^CD8+ T cells were detected in significantly higher proportion in PR (median 13.62%; range 3.77 to 26.91%) than in NP (median 0.34%; range 0 to 25.78%) and ART-treated individuals (median 4.31%; range 0 to 28.15%). Surprisingly, the proportion of responding CD45RA+ IFN-γ^neg ^IL-2^neg ^MIP-1β+ CD8+ T cells in NP was significantly higher than in PR and ART-treated patients with extremely low p values (p = 0.0067 and p = 0.0002, respectively; Figure 2B and 2C). Indeed, CD45RA+ IFN-γ^neg ^IL-2^neg ^MIP-1β+ responding CD8+ T cells were detected in 7 out of 11 NP (64%) and 1 out of 10 PR (10%), whereas they were completely undetectable in the 22 ART-treated patients analyzed (Nef-specific responses were not analyzed in subject ART07). Interestingly, Nef-specific CD45RA+ IFN-γ^neg ^IL-2^neg ^MIP-1β+ CD8+ T cells in NP, when detectable, represented a high proportion of the total response (range: 10.7 to 49.9%). The same population detected in one PR (PR05) represented only 6.8% of the total response. As shown in figures 2C and 2D, the strong association between CD45RA+ IFN-γ^neg ^IL-2^neg ^MIP-1β+ CD8+ T cells and the NP cohort was a distinctive feature of this MIP-1β+ cell population and was not shared with other MIP-1β+ T-cell populations. In fact, although a significant higher proportion of CD45RA+ IFN-γ+ IL-2^neg ^MIP-1β+ responding CD8+ T cells was observed in NP in comparison to ART-treated individuals (p = 0.0324; Figure 2B and 2C), CD45RA+ IFN-γ+ IL-2^neg ^MIP-1β+ CD8+ T cells were detectable in PR and ART-treated individuals and were not uniquely associated with the NP cohort. Similarly, CD45RA^neg ^Nef-specific CD8+ T cells either IFN-γ^neg ^IL-2^neg ^MIP-1β+ or IFN-γ+ IL-2^neg ^MIP-1β+ were detected in high frequencies in all the three cohorts (Figure 2B and 2D).

**Figure 3:**
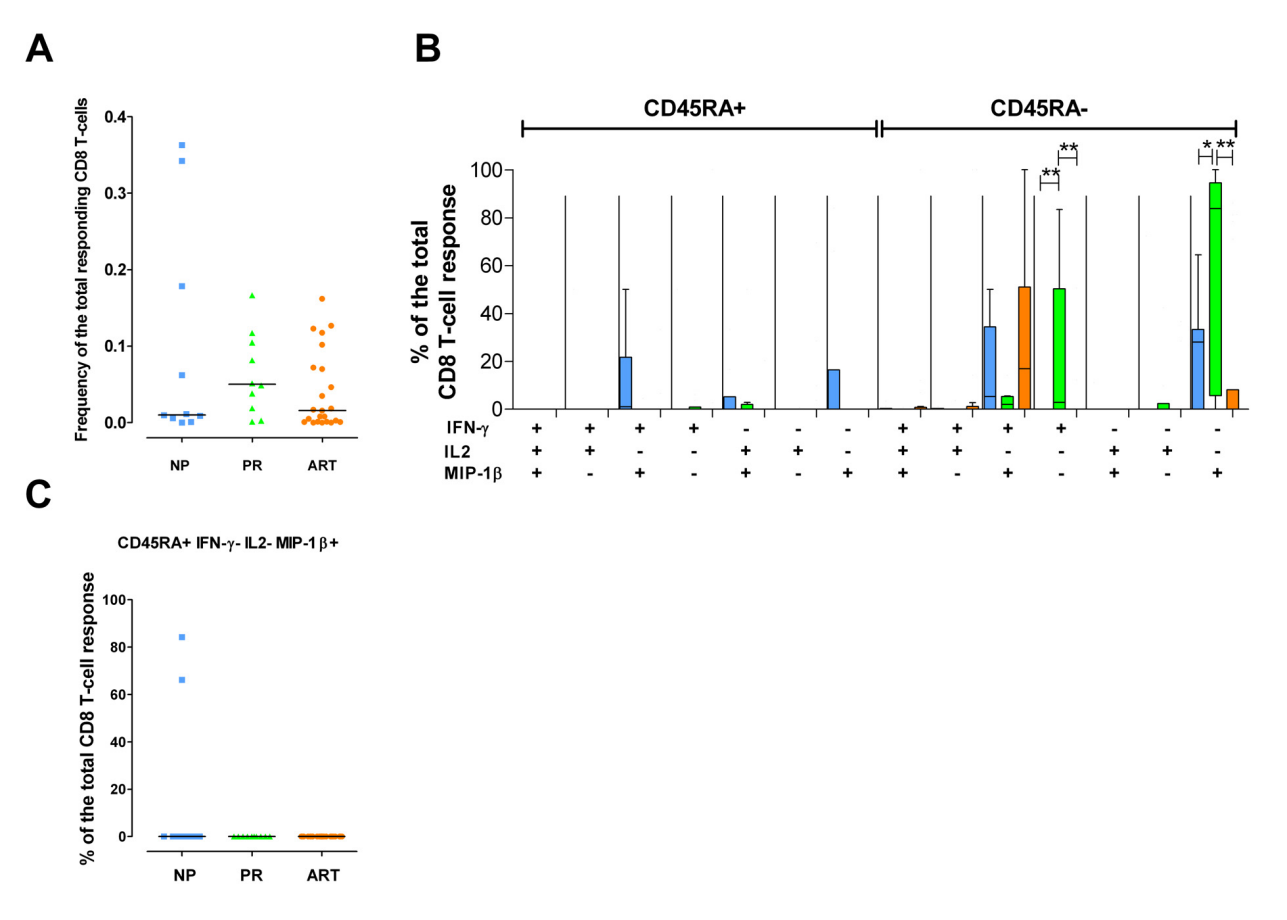
**HIV-1-Tat-specific CD8+ T-cell responses**. HIV-1-Tat-specific CD8+ T-cell responses in 10 NP (blue), 10 PR (green) and 23 ART-treated patients (orange). Tat-specific responses were not analyzed in NP11. (**A**) Frequency of the total Tat-specific CD8+ T cells. (**B**) Quality of the Tat-specific response. The graph is divided into the CD45RA+ (left part) and the CD45RA^neg ^(right part) CD8+ T-cell populations. All the possible combinations of the responses are shown on the x-axis for NP, PR and ART-treated individuals. Tukey boxes and whisker plots are shown. Significant differences are noted above the graph: (*) p < 0.05, (**) p < 0.01 and (***) p < 0.001. **(C) **Individual data point representation of the Tat-specific CD45RA+ IFN-γ^neg ^IL-2^neg ^MIP-1β+ CD8+ T-cells. In all graphs, medians are represented by horizontal bars.

In comparison to Nef-specific responses, Tat-specific CD8+ T cells were characterized by lower magnitude and, worthy of note, no significant differences were observed in the total CD8+ T-cell responses among the cohorts analyzed (Figure 3A). Significantly higher proportions of Tat-specific CD45RA^neg ^IFN-γ+ IL-2^neg ^MIP-1β^neg ^and CD45RA^neg ^IFN-γ^neg ^IL-2^neg ^MIP-1β+ CD8+ T cells were observed in NP than in PR and ART-treated individuals (Figure 3B). Of note, CD45RA+ IFN-γ^neg ^IL-2^neg ^MIP-1β+ responding CD8+ T cells were found in 2 out of 10 NP that showed Tat-specific CD8+ T-cell responses, while none of the remaining Tat responders in the other cohorts showed this cell population (Figure 3C).

Overall, we observed that monofunctional CD8+ T cells were prevalent in PR whereas polyfunctional CD8+ T cells were prevalent in individuals in whom the viral load was kept under control either naturally or with the help of antiretroviral treatment. Of particular interest, we identified a novel Nef-specific CD45RA+ IFN-γ^neg ^IL-2^neg ^MIP-1β+ CD8+ T-cell population specifically associated with prolonged spontaneous control of HIV-1 disease progression in the absence of ART.

### Nef- and Tat-specific CD45RA+ IFN-γ^neg ^IL-2^neg ^MIP-1β+ CD8+ T cells are not driven by viral load

We next explored the potential effect of differences in the level of viremia on the presence of CD45RA+ IFN-γ^neg ^IL-2^neg ^MIP-1β+ responding CD8+ T cells in NP. Our cohort of NP was characterized by a prolonged exposure to HIV-1 antigens since their seropositivity was diagnosed with a median of 19 years (range: 9-25). In addition, NP showed detectable plasma viremia, although at low levels (range: 50-10,756 RNA copies/ml). As a consequence, antigen exposure could have played a direct role in generating CD45RA+ IFN-γ^neg ^IL-2^neg ^MIP-1β+ CD8+ T cells. However, the analysis of the relationship between plasma viremia and Nef-specific CD45RA+ IFN-γ^neg ^IL-2^neg ^MIP-1β+ CD8+ T cells expressed as percentage of the total Nef-specific response or as percentage of the total CD8+ T cells revealed no significant correlation (data not shown). Furthermore, only one PR (10%) showed detectable levels of Nef-specific CD45RA+ IFN-γ^neg ^IL-2^neg ^MIP-1β+ CD8+ T cells, supporting the idea that this novel CD8 T-cell population is not directly driven by antigen levels.

**Figure 4:**
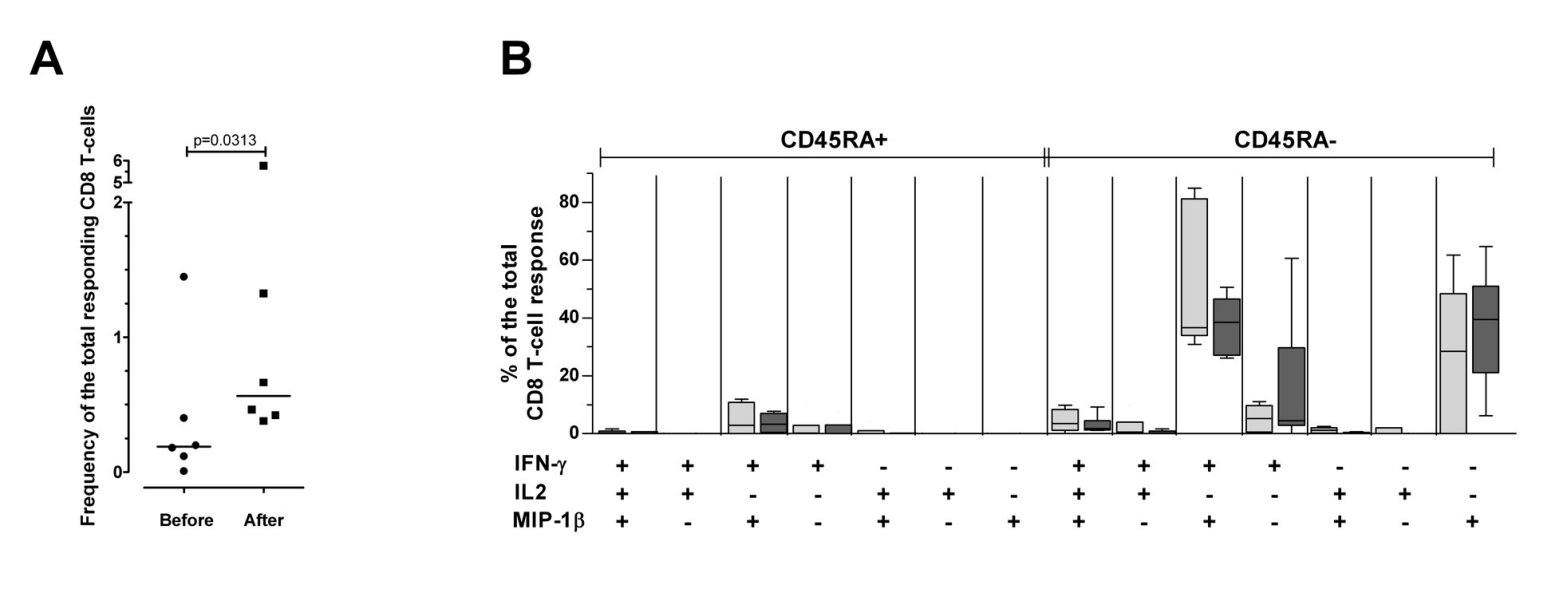
**Nef-specific CD8+ T-cell responses during TI**. (**A**) Frequency of the total Nef-specific response before and after TI. The median is shown for each group. (**B**) Quality of the Nef-specific CD8+ T-cell responses before (light gray boxes) and after (dark gray boxes) TI. The graph is divided into the CD45RA+ (left part) and the CD45RA^neg ^(right part) CD8+ T-cell populations. All possible combinations of responses are shown on the X axis. Tukey boxes and whisker plots are shown.

To investigate further the role of *in vivo *HIV-1 replication in generating CD45RA+ IFN-γ^neg ^IL-2^neg ^MIP-1β+ CD8+ T cells, we analyzed Nef- and Tat-specific CD8+ T-cell responses in a longitudinal set-up. Six ART-treated patients with highly suppressed viremia (ART01, ART02, ART03, ART04, ART05 and ART06) underwent a single cycle of therapy interruption (TI). Viremia became detectable in all patients between day 5 and 21 after TI. ART was resumed between day 27 and 185 when viremia levels reached >100,000 HIV-1 RNA copies/ml. A significant expansion of the Nef-specific CD8+ T-cell responses was observed in all the subjects analyzed (Figure 4A). However, the quality of the CD8+ T-cell response remained unchanged in that CD45RA+ IFN-γ^neg ^IL-2^neg ^MIP-1β+ CD8+ T cells remained undetectable even during the boost of the total Nef-specific CD8+ T-cell response that followed the peak of virus replication post TI (Figure 4B). Of note, we observed a decrease of Nef-specific CD8+ T cells expressing multiple effector functions and an increase of Nef-specific CD8+ T cells expressing solely IFN-γ, but these differences were not significant, probably due to the low number of subjects included in the longitudinal analysis. The Tat-specific CD8+ T-cell response was substantially undetectable before and after TI (data not shown).

Thus, in our experimental setting CD45RA+ IFN-γ^neg ^IL-2^neg ^MIP-1β+ CD8+ T cells did not appear after induction of strong *in vivo *viral replication.

### IL-2 is not an essential marker to define the exclusive detection of CD45RA+ IFN-γ^neg ^IL2^neg ^MIP-1β+ CD8+ T cells in NP

Since in our cohorts of HIV-1 infected patients IL-2-producing cells were rarely detected, we reanalyzed the data shown in Figure 2B considering only the combined expression of CD45RA, IFN-γ and MIP-1β. The proportion of responding CD45RA+ IFN-γ^neg ^MIP-1β+ CD8+ T cells was significantly higher in NP than in PR and ART-treated patients (p = 0.0069 and p = 0.0012, respectively). This observation indicates that IL-2 expression represents neither an essential marker of nonprogressive HIV-1 infection nor a distinctive feature of CD45RA+ IFN-γ^neg ^MIP-1β+ CD8+ T cells in NP.

### CD45RA+ IFN-γ^neg ^MIP-1β+ CD8+ T cells are truly terminally differentiated effector CD8+ T cells

**Figure 5:**
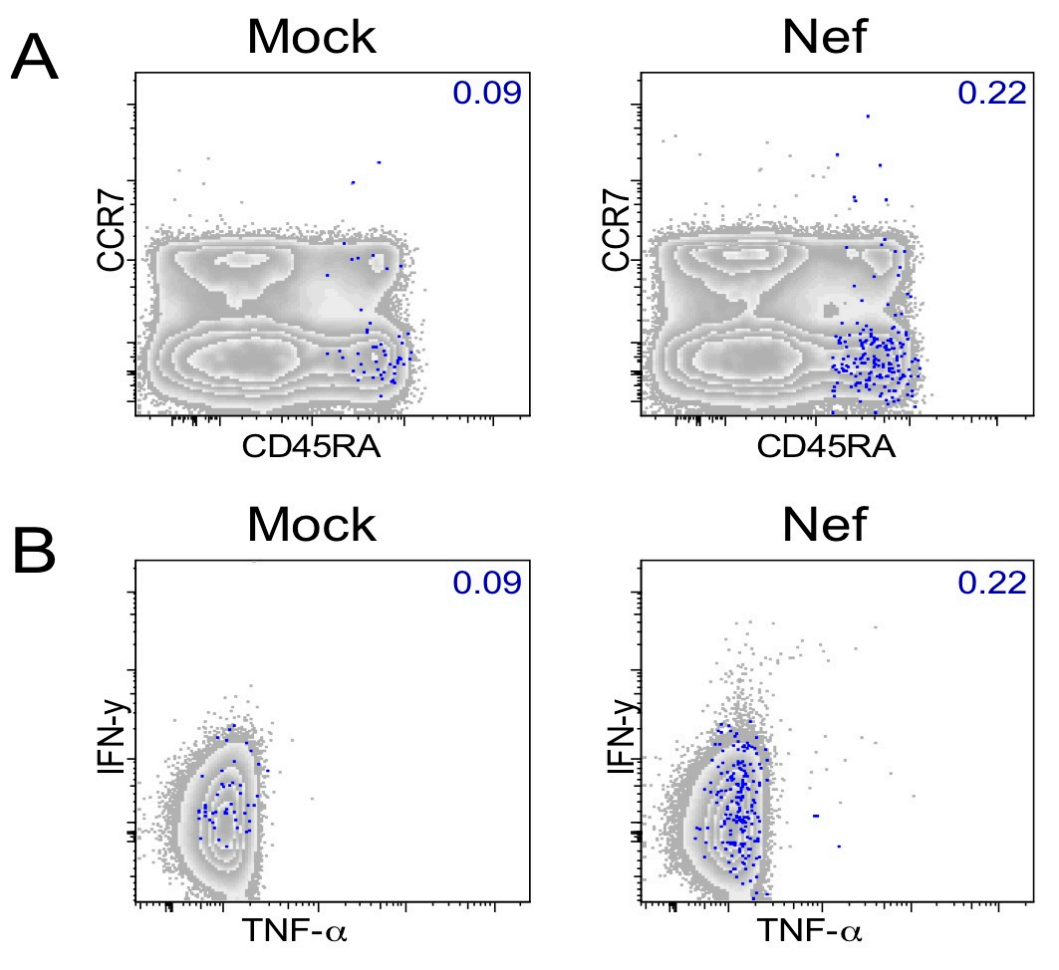
**Characterization of Nef-specific CD45RA+ IFN-γ**^**neg **^**MIP-1β+ CD8+ T cells**. Total CD8+ T cells of patient V4 are shown according to CD45RA/CCR7 (**A**) and TNF-α/IFN-γ (**B**) expression. Grey zebra plots show total CD8+ T cells. Percentages of CD45RA+ IFN-γ^neg ^MIP-1β+ CD8+ T cells, shown as blue dots are indicated in each graph. Mock-stimulated control samples are shown in the left and Nef-stimulated samples in the right panels.

The use of the sole CD45RA marker cannot discriminate between experienced and naive T-cells, when not associated with other cellular markers such as CCR7 or CD27. However, in the present study, we analyzed cells able to produce cytokines or chemokines following a short (5 hours) antigenic peptide stimulation. Thus, only experienced (memory or effector T-cells) can be detected by this assay, since the number of circulating naive T-cells carrying a T-cell receptor specific for a given peptide is too low to be detected by short term assays. Nevertheless, we characterized the expression of CCR7 in CD45RA+ IFN-γ^neg ^MIP-1β+ CD8+ T cells derived from one HIV-1 infected individual that following treatment interruption showed a partial control of viral replication (patient V4, see Materials and Methods). In this patient, Nef-specific CD45RA+ IFN-γ^neg ^MIP-1β+ CD8+ T cells were previously characterized (unpublished data). As shown in Figure 5A, Nef-specific CD45RA+ IFN-γ^neg ^MIP-1β+ CD8+ T cells were CCR7^neg^. In the same experiment, we additionally measured TNF-α expression and observed that Nef-specific CD45RA+ IFN-γ^neg ^MIP-1β+ CD8+ T cells did not express TNF-α upon antigenic peptide stimulation (Figure 5B).

In conclusion, CD45RA+ IFN-γ^neg ^MIP-1β+ CD8+ T cells did not express the chemokine receptor CCR7 and are therefore classified as effector CD8+ T-cells (Figure 5A). Furthermore we determined that CD45RA+ IFN-γ^neg ^MIP-1β+ CD8+ T cells did not produce TNF-α in patient V4 (Figure 5B).

## Discussion

A fundamental prerequisite for the development of immune-based therapies and an effective vaccine against HIV/AIDS is the identification of solid immune correlates of disease progression. In order to identify such correlates, we compared Nef and Tat specific CD8+ T-cell immune responses in three cohorts of HIV-1 infected individuals with different degree of HIV-1 control: NP, PR and ART-treated patients. With this setting, we identified a novel population of CD8+ T cells associated with nonprogressive HIV-1 infection. CD45RA+ IFN-γ^neg ^MIP-1β+ CD8+ T cells that we henceforth entitle MIRA (**MI**P-1β+ CD45**RA**+) CD8+ T cells potentially represent a valuable immune correlate of disease progression, since they were detected in response to Nef stimulation in 7 out of 11 NP, in only 1 out of 10 PR and were completely absent in 22 ART-treated patients. We further demonstrated on 6 ART-treated patients undergoing single cycle TI that the presence of MIRA CD8+ T cells is independent of viral load. These observations render this novel population particularly interesting as a potential surrogate clinical marker of immunological reconstitution or maintenance after immune-based interventions.

According to the expression of CD45RA and CCR7, antigen-experienced CD8+ T cells are classified as T_CM _(CD45RA^neg ^CCR7+), T_EM _(CD45RA^neg ^CCR7^neg^) or T_EMRA _(CD45RA+ CCR7^neg^) [22]. Since MIRA CD8+ T cells express CD45RA and secrete MIP-1β upon specific antigenic stimulation, they likely belong to the T_EMRA _population. The absence of CCR7 expression by MIRA CD8+ T cells was demonstrated in one representative sample, further supporting the T_EMRA _phenotype. Several studies have suggested a possible role of the fully differentiated HIV-1-specific T_EMRA _CD8+ T cells in the effective control of HIV-1 replication: IFN-γ producing T_EMRA _CD8+ T cells have been associated with the control of virus replication in NP [14] and in individuals with early infection and low viral set point thereafter [15]. Furthermore, antigen-specific T_EMRA _CD8+ T cells were preferentially detected in acutely infected individuals who achieved control of viremia either spontaneously or after structured TI [19]. A pre-terminally differentiation status or skewed maturation phenotype (mainly composed by T_EM _cells) has been reported for HIV-1-specific CD8+ T cells in therapy-naive viremic patients [23,24]. The skewed maturation of HIV-1-specific CD8+ T cells in comparison to other better controlled persistent infections has been considered as a defective immune response. Altogether, these studies indicate an important role of terminally differentiated CD8+ T cells in the control of HIV-1 replication *in vivo*. Here we support and extend the link between HIV-1-specific T_EMRA _CD8+ T cells and slow disease progression by identification of a novel HIV-1-specific population of effector cells specific for nonprogressive HIV-infection.

Polyfunctional CD8+ T cells have been previously described in NP [13]. This observation is consistent with our study, in which the proportion of responding CD45RA+ IFN-γ+ IL-2+ MIP-1β+ CD8+ T cells was significantly higher in NP than in ART-treated individuals (p = 0.0067; Figure 2B). In addition, we observed higher proportions of polyfunctional CD45RA^neg ^CD8+ T cells in ART-treated individuals in comparison to PR (Figure 2B) and higher proportions of monofunctional (IFN-γ+ IL-2^neg ^MIP-1β^neg^) CD45RA^neg ^CD8+ T cells in PR in comparison to NP and ART-treated individuals (Figure 2B). These data support the idea that polyfunctional CD8+ T cells are lost during progressive HIV-1 replication and are maintained or recovered during nonprogressive infection or treatment with ART. A recent longitudinal study demonstrated that polyfunctional CD8+ T cells re-emerge following prolonged ART-mediated viral suppression [16]. Furthermore, Streeck et al. [25] demonstrated that monofunctional HIV-1-specific CD8+ T cells decrease upon removal of antigenic stimulation. Together with these previous studies, our data suggest that persistent stimulation by antigen can cause functional CD8+ T-cell impairment and may lead to enrichment of monofunctional IFN-γ producing HIV-1-specific CD8+ T cells.

In chronic viral infections, the main obstacle to the definition of a correlate of disease progression is the ability to discriminate between phenotypes responsible for the control of viral replication and phenotypes that are the consequence of a different infection history [16]. MIRA CD8+ T cells were undetectable in a group of 4 ART-treated patients with a previous history as NP (Table 1 and Materials and Methods), suggesting that this cell population is absent when patients lose the capacity to control virus replication. The absence of MIRA CD8+ T cells in 9 out of 10 PR and 3 ART-treated patients with detectable viremia together with the analysis of a group of 6 ART-treated patients undergoing a single cycle of TI demonstrated that MIRA CD8+ T cells are not induced or regulated by the *in vivo *levels of HIV-1 replication. In this regard, no correlation was found between the proportion of MIRA CD8+ T cells and the levels of viremia in the 11 NP analyzed, even though the subject with the highest viremia (NP13) showed also the highest total Nef-specific response with a large proportion of Nef-specific MIRA CD8+ T cells (36.5%). These observations suggest that MIRA CD8+ T cells are not the direct consequence of ongoing *in vivo *antigen exposure, but possibly may represent a correlate of HIV-1 disease progression. However, it can be suggested that the prolonged exposure to low levels of plasma viremia in NP is responsible for the appearance of MIRA CD8+ T cells, and that MIRA CD8+ T cells do not appear following the short term exposure to high level of viremia during TI. To address this hypothesis it would be helpful to analyze elite controllers, who are characterized by controlled HIV infection with undetectable viral load. In addition, more detailed longitudinal studies will be necessary to definitively demonstrate the role of MIRA CD8+ T cells in HIV-1 infection.

Several reports showed an exceptional HLA class I associated sequence polymorphism in the *nef *and *p24 *genes in comparison to other HIV-1 genes [26,27], suggesting a strong selective pressure exerted by CD8+ T cells targeting Nef and p24. Data from SIV Nef vaccinated macaques provided evidence that Nef-specific CD8+ T cells might contribute to the control of SIV infection [28]. Our study was limited to the analysis of the Nef and Tat-specific CD8+ T-cell responses since these two genes represent candidate HIV-1 vaccines within the AIDS Vaccine Integrated Project (AVIP, http://www.avip-eu.org. A genome-wide analysis of the HIV-1-specific response using our intracellular cytokine staining analysis will aid in understanding the role of MIRA CD8+ T cells specific to p24 and other HIV-1 antigens in the control of viral replication.

Our study demonstrated that the sole measurement of the Nef-specific response might be sufficient to define MIRA CD8+ T cells as a correlate of nonprogression in HIV-1 disease. In addition, the presence of MIRA CD8+ T cells in 2 Tat-responding NP and the absence of the same population in the Tat-responding PR and ART-treated patients suggest that MIRA CD8+ T cells may represent a correlate of nonprogression independently of the targeted viral protein. Since, it has been described that the use of autologous peptides allows the detection of stronger and broader T-cell responses [29], we cannot exclude that the use of a set of autologous peptides or the use of a consensus sequence would have increased the detection of Tat-specific responses. Further studies should address which kind of sequence will better suit to detect MIRA CD8+ T cells specific to different HIV-1 proteins.

HIV-1 infection causes hyperactivation of the immune system leading to immune exhaustion and disease progression [30-32]. Since MIRA CD8+ T cells produce neither IFN-γ nor IL-2, it can be suggested that due to their limited effector function they do not contribute to hyperactivation in chronic HIV-1 infection. In contrast, in early infection, the effector function of CD8+ T cells is essential in controlling the initial viral replication [33]. Thus, the limited functionality of MIRA CD8+ T cells may contribute to rapid progression in the early stage of infection. This could explain why MIRA CD8+ T-cells were detected in progressor PR05, the only patient within the PR cohort who presented with the clinical phenotype of a rapid progressor.

Since the present study was observational, it was not our objective to clarify whether MIRA CD8+ T cells exert a direct protective function. However, it has been shown, that MIP-1β dominates HIV-1-specific CD8+ T-cell responses [13,34] and that high levels of MIP-1β are associated with decreased risk of progression to AIDS [35]. Furthermore, MIP-1β is a potent natural inhibitor of CCR5-mediated HIV-1 entry [36]. IFN-γ was shown to be capable to upregulate HIV-1 replication [37,38] and to induce the expression of HIV in persistently infected cells in culture [39]. We can therefore speculate that in the absence of IFN-γ, MIP-1β secreted by MIRA CD8+ T cells provide HIV-1 inhibitory functions.

## Conclusion

In conclusion, our study presents a novel population of Nef-specific effector CD8+ T cells associated with nonprogressive HIV-1 infection. This population, named MIRA, expresses CD45RA and produces MIP-1β but not IFN-γ. This T cell subset was shown to be independent of *in vivo *viral replication. MIRA CD8+ T cells may be useful to ameliorate the timing of ART initiation in HIV-1 infected individuals and represent a potential correlate to determine the efficacy of immune-based interventions.

## Materials and methods

### Patients

Eleven NP, 10 PR and 23 ART-treated patients were included in the present study. Of the 11 NP subjects, 10 matched the definition of long-term nonprogressors (LTNP), i.e. naive to ART with a documented HIV-1 infection of >9 years (median: 19.5 years), CD4+ T-cell counts ranging between 421 and 1,042 (median: 522 cells/μl). In comparison to the other NP, study subject NP11 had lower levels of CD4+ T-cell counts (274 cells/μl). Nevertheless he was included in the NP cohort because of 19 years of documented history of HIV-1 infection, with stable CD4+ T-cell counts over a one year follow-up post-sampling (range: 256-310 cells/μl). The median plasma viral load in the 11 NP was 900 HIV-1 RNA copies/ml (range: <50-10756). PR had poor restriction of viral replication (HIV-1 RNA copies/ml >99000) and declining CD4+ T-cell counts (median: 303 cells/μl; range: 132-474). All the PR were ART naive. PR05 was the only rapid progressor in the PR cohort. Already five month after putative HIV-1 infection (the timepoint of blood sampling) CD4+ T-cell counts dropped in this patient to 208 counts/ml without recovery and persistently high level viremia. The 23 ART-treated patients were on treatment for 2 or more years. Twenty ART-treated subjects had undetectable viral load, whereas subjects ART12, ART11 and ART23 had 36600, 3362 and 13965 HIV-1 RNA copies/ml, respectively. The median CD4+ T-cell count in the 23 ART-treated subjects was 488 cells/μl (range: 229-969). Study subjects ART12, ART14, ART15 and ART16 were previously classified as NP but initiated ART more than 4 years before blood sampling for the present study and therefore are here included in the ART-treated cohort. Six of the ART-treated individuals (ART01, ART02, ART03, ART04, ART05 and ART06) underwent a single cycle of therapy interruption (TI). From these patients PBMC were obtained before TI (Table 1) and at the peak of the immune response after TI. Viremia was monitored weekly and treatment restarted at the first viral load determination >100,000 HIV-1 RNA copies/ml. Patients remained off treatment for a median of 31 days (range: 26-182 days). HIV-1 infected patient V4 was due to his clinical characteristics not member of any of the above described cohorts. At the timepoint of blood sampling (3 years after TI) viral replication was controlled to 48600 HIV-1 RNA copies/ml. CD4 T-cell counts before and after blood sampling were 758 and 871 cells/μl, respectively.

The study was approved by the local Institutional Review Boards (Comitato Etico - Ospedale San Raffaele; Ethic Commitee of the Azienda Ospedaliera della Provincia di Lodi; Ethikkommission der Medizinischen Fakultät der Ludwig-Maximilians-Universität München; Ethikkommission der Ärztekammer Hamburg). Written informed consent was obtained for all study participants.

### Antibodies

CD3-AmCyan, CD4-PerCP, CD45RA-PE-Cy7, CD154-FITC, IFNγ-Al700, IL2-APC and MIP-1β-PE were obtained from Becton Dickinson. CD8-PacB was obtained from DAKO. CCR7-FITC and TNFα-PacB were obtained from R&D and NatuTec, respectively.

### Antigens

Nef- and Tat-specific cellular responses were identified by using two sets of overlapping peptides covering the HIV-1 encoded Tat (BH10 strain) and Nef (BRU strain) antigens. Nef peptides were divided into 2 pools: Nef N-term (amino acids 1 to 101) and Nef C-term (amino acids 97 to 206). All pools were used at the final concentration of 2 μg/ml of each peptide. The Tat- and Nef-specific sets of overlapping peptides have been designed using a strategy termed Variable Overlapping Peptide Scanning Design (VOPSD) [40] that has been validated within the EU-sponsored AVIP consortium by a ring trial involving 5 different European and South African laboratories.

Briefly, all peptides were biased at the C-terminus avoiding 7 amino-acid residues never found among optimal CTL epitopes and MHC class I binding motifs (Asn, Asp, Gln, Glu, Gly, His, Ser). Optimal epitopes were placed in the N- or C-terminus of the peptides. Finally, conserved and variable regions were segregated in different peptides. In order to fit all the above mentioned design features the derived peptides were conceived to be variable in length (10 to 19 amino-acids) and degree of overlap (5 to 14 amino-acids, with a gap ≤8 amino-acids). Moreover, the degree of overlap and the overall peptide length were empirically tuned according to the frequency of known T-cell epitopes (higher overlap and shorter peptide length in epitope-rich regions and, conversely, wider not-overlapping fragment and maximal peptide length in epitope-poor regions). Peptides were obtained through the Centre for AIDS Reagents, National Institute for Biological Standards and Control, Hertfordshire, UK.

### Intracellular cytokine staining

Cryopreserved samples were used throughout the study. The 9-color intracellular cytokine staining used in the present study has been previously described [34]. Briefly, all the incubations were carried out in round-bottom 96-well plates to allow high-throughput processing of the probes. 10^6 ^PBMC per well were incubated with 1.3 μg/ml of anti-CD28 mAb and with 1.3 μg/ml of anti-CD49d mAb (Becton Dickinson) together with defined peptide pools. For each individual donor, a sample without peptides was included to calculate the background staining. Following 60 min incubation, 10 μg/ml of Brefeldin A (Sigma) were added to the cell suspension and the incubation was carried out for additional 4 h. Stimulated cells were then resuspended in Stain Buffer (0.2% BSA, 0.09% Na Azide in Dulbecco's PBS; Becton Dickinson) and incubated with the photoreactive fluorescent label ethidium monoazide (EMA; Molecular Probes) to assess their viability. After washing, cells were fixed and permeabilized using the BD Cytofix/Cytoperm™ Kit (Becton Dickinson). Then fluorochrome-conjugated Abs were added to the cell suspension. Incubation was carried out on ice for 30 min and after washing, cells were acquired using an LSRII flow cytometer (Becton Dickinson) equipped with a high throughput system. Sample analysis was performed using FlowJo version 8 (Tree Star). Lymphocytes were gated on a forward scatter area versus side scatter area pseudo-color dot plot and dead cells were removed according to EMA staining. Events were gated on CD3+ events versus IFN-γ, IL-2, MIP-1β and CD154 to account for down-regulation. CD3+ events were then combined together using the Boolean operator "OR". The same procedure was used to subsequently gate CD8+ events. CD4+ events were excluded before creating a gate for each function or phenotype as shown in Figure 1. In consistency with others [41,13,42] positive events were determined by gating on combinations of markers that provided optimal separation between positive and negative populations. Each gate selected all cells positive for the marker shown on the x-axis, while the marker chosen on the y-axis was selected to provide a better separation between positive and negative events. By calculating every possible combination of the 5 populations gated in Figure 1, Boolean gating analysis identified 32 response patterns. Response patterns with at least 1 positive functional marker were taken into consideration for analysis, thus resulting in a total of 30 immune response patterns.

### Statistical analysis

Since background levels varied between subpopulations, i.e. MIP-1β staining showed a higher background than IFN-γ, and the combination of 3 or more functions had extremely low background, we calculated a threshold level for each subpopulation. Following background subtraction, the threshold level was calculated using the 90th percentile of the negative values. Then, all the resulting negative values were set to zero and all values >0 were considered as positive responses. The frequency of the total CD8+ T cell response was calculated by summing each unique CD8+ T-cell population expressing at least one functional marker. Percentages of total responses were calculated by dividing the frequency of each subpopulation by the frequency of the total CD8+ T cell response. In order to analyze the quality of the response without considering the CD154 marker, the CD154+ and CD154^neg ^CD8+ T-cell populations were combined using SPICE software. SPICE version 4.1.5 (Mario Roederer, Vaccine Research Center, National Institute of Allergy and Infectious Diseases, National Institutes of Health) and Prism version 5.01 (GraphPad Software) were used for graphical representation of the data and statistical analysis. To compare paired groups, the Wilcoxon signed-rank test was performed. To account for multiple comparisons, a closed testing procedure was applied in testing differences of all groups by the Kruskal-Wallis test followed by pair-wise comparisons with the Wilcoxon rank sum test. The SAS Version 9.1 was used to get exact P values for the statistical analysis.

## Competing interests

AC, PL, GP, VE and MM are named as inventors on a provisional patent application based on this work. The patent has been filled by the Helmholtz Zentrum München - German Research Center for Environmental Health and the San Raffaele Scientific Institute.

## Authors' contributions

GP, VE, MM and AC conceived the study. CJD, SK, SA and AC established the flow cytometry based assays. CJD, SK, SH, SA and DH performed experiments. SG, EV, GT, JRB, HJS, ES, TV, FDG, RD, MT, GP and AC participated to the collection of patient samples. CJD, SH, SK, SA, PB, PR, MM and AC analyzed data. PL and UP contributed to research and critical discussion. AC wrote the paper. All authors read and approved the final manuscript.
